# Directed Evolution of an Angiopoietin-2 Ligand Trap by Somatic Hypermutation and Cell Surface Display*

**DOI:** 10.1074/jbc.M113.510578

**Published:** 2013-10-08

**Authors:** Nicholas P. J. Brindle, Julian E. Sale, Hiroshi Arakawa, Jean-Marie Buerstedde, Teonchit Nuamchit, Shikha Sharma, Kathryn H. Steele

**Affiliations:** From the Departments of ‡ Cardiovascular Sciences and; § Biochemistry, University of Leicester, Leicester LE1 9HN, United Kingdom,; the ¶ Division of Protein and Nucleic Acid Chemistry, Medical Research Council Laboratory of Molecular Biology, Cambridge CB2 0QH, United Kingdom,; the ‖ Italian Foundation for Cancer Research Institute of Molecular Oncology, European Institute of Oncology, 20139 Milano, Italy, and; the ** Department of Immunobiology, Yale University School of Medicine, New Haven, Connecticut 06520

**Keywords:** Angiogenesis, Cell Surface Receptor, Directed Evolution, Endothelial Cell, Ligand-binding Protein, Protein Engineering

## Abstract

Tie2 is a receptor tyrosine kinase that is essential for the development and maintenance of blood vessels through binding the soluble ligands angiopoietin 1 (Ang1) and 2 (Ang2). Ang1 is constitutively produced by perivascular cells and is protective of the adult vasculature. Ang2 plays an important role in blood vessel formation and is normally expressed during development. However, its re-expression in disease states, including cancer and sepsis, results in destabilization of blood vessels contributing to the pathology of these conditions. Ang2 is thus an attractive therapeutic target. Here we report the directed evolution of a ligand trap for Ang2 by harnessing the B cell somatic hypermutation machinery and coupling this to selectable cell surface display of a Tie2 ectodomain. Directed evolution produced an unexpected combination of mutations resulting in loss of Ang1 binding but maintenance of Ang2 binding. A soluble form of the evolved ectodomain binds Ang2 but not Ang1. Furthermore, the soluble evolved ectodomain blocks Ang2 effects on endothelial cells without interfering with Ang1 activity. Our study has created a novel Ang2 ligand trap and provided proof of concept for combining surface display and exogenous gene diversification in B cells for evolution of a non-immunoglobulin target.

## Teaser

**Background:** The ligand angiopoietin2 contributes to vascular diseases.

**Results:** A new directed evolution method was used to create a specific angiopoietin2-binding protein from a nonspecific angiopoietin receptor.

**Conclusion:** The receptor binding specificity can be switched with just three residue changes.

**Significance:** The new protein has therapeutic potential, and the directed evolution method has advantages for evolving mammalian proteins.

## Introduction

Angiopoietin-2 (Ang2)^2^ is a 70-kDa secreted ligand whose increased expression has been implicated in a range of diseases, including cancer, sepsis, and acute respiratory distress syndrome (1, 2). The primary receptor for Ang2 is the transmembrane tyrosine kinase Tie2 (3) that is expressed mainly on vascular endothelial cells and myeloid cells (1, 4). Ang2 plays an important role in vascular remodeling during development, but in adult tissues, Ang2 concentrations are usually low. An increase in Ang2 levels in disease allows the molecule to compete for binding to a common interface on Tie2 with the related agonist Ang1 (3). Ang1 is a protective protein, constitutively produced by perivascular cells, which maintains blood vessel function and quiescence by suppressing inflammation, vessel leakage, and endothelial apoptosis (1, 5). Antagonism of Ang1 by Ang2 blocks the proquiescent effects of Ang1 and contributes to Ang2-induced vessel remodeling, inflammation, leakage, and edema. In addition to its actions on endothelial Tie2, Ang2 has a number of other effects relevant to disease. For example, the ligand acts on tumor infiltrating Tie2-expressing monocytes to promote tumorigenesis (6, 7).

Because of its involvement in multiple disease processes, there have been considerable efforts to develop inhibitors of Ang2, including antibodies and aptamers (8–10). Results from studies with these and related molecules have been encouraging, with reports of Ang2 inhibitors promoting tumor regression and suppressing metastatic disease in cancer and decreasing leukocyte infiltration and vascular remodeling in airway inflammation (6, 9, 11, 12).

A complementary approach to the use of antibodies for blocking pathological levels of ligands is the cytokine or ligand trap (13). These molecules are formed from receptor ectodomain fragments, usually administered as soluble fusion proteins, which sequester the target ligand. Examples of ligand traps in clinical use include Etanercept, a soluble form of tumor necrosis factor-α receptor, and Aflibercept, a chimeric fusion protein of fragments of vascular endothelial growth factor receptor-1 and -2 (14). There are significant advantages to ligand traps. Usually, they are smaller and have better tissue penetration than antibodies, they already recognize the biologically active part of the target, and they generally do not require protection from the immune system. A ligand trap specific for Ang2 would be an attractive therapeutic; however, the natural receptor for Ang2, Tie2, binds to the protective ligand Ang1 equally well or even better than it does to Ang2 (3, 15, 16). We were interested therefore to try and engineer a variant form of Tie2 ectodomain that would preferentially bind Ang2 over Ang1. Such a molecule would be an important starting point for development of a therapeutic Ang2 ligand trap that could be used to block the damaging effects of this ligand without suppressing the protective effects of Ang1.

One of the most effective strategies for engineering new protein functionality is directed protein evolution (17, 18). This process essentially recapitulates the selection and accumulation of desirable mutations that occurs in natural evolution over millions of years, but over a period of weeks in the laboratory. Directed evolution involves repeated rounds of library construction, usually *in vitro*, expression of the mutant forms of the target protein, and selection. Unfortunately, this iterative approach to *in vitro* generation and searching of sequence space is frequently both difficult and labor-intensive. B cell lines that constitutively diversify their immunoglobulin variable (IgV) regions by somatic hypermutation (19) allow for facile coupling of diversification and selection of novel antibody specificities because the genetic variation within the Ig genes, introduced by the action of activation-induced deaminase, is coupled to the selectable expression of surface Ig on individual cells (20). Such cell lines have been used to evolve variants of green fluorescent protein exogenously expressed within the cells (21, 22). However, in theory, this strategy has enormous potential for directed evolution of a wide range of proteins if the desired phenotype can be selected for in B cell lines. To date, this approach has not been applied to non-immunoglobulin cell surface proteins. Here we report successful combination of cell surface display on B cells together with somatic hypermutation-driven gene diversification to evolve a form of Tie2 ectodomain with preferential binding to Ang2. This switch in binding specificity of the ectodomain resulted from just three amino acid changes. The evolved ectodomain acts as an Ang2 ligand trap and has potential for therapeutic blocking of Ang2 in a number of diseases.

## EXPERIMENTAL PROCEDURES

### 

#### 

##### Materials

cDNA encoding human Tie2 ectodomain (1–442) and platelet-derived growth factor receptor β (residues 514–562, which includes the transmembrane sequence), with an amino-terminal five alanine linker followed by the FLAG epitope, were generated by polymerase chain reaction. These amplification products were ligated into pcDNA3.1 and then transferred to the vector pHypermut2 (22). All constructs were verified by sequencing. Human Ang1, Ang2, biotinylated Ang2, and mouse Anti-Ang1 were obtained from R&D Systems. Anti-FLAG conjugated to FITC and streptavidin conjugated to phycoerythrin or phycoerythrin/Cy5 were from Sigma, and anti-His_6_ conjugated to allophycocyanin was from Abcam. Goat anti-mouse conjugated to Percp/Cy5.5 was from Biolegend. Antibodies recognizing Akt and phospho-Ser-473-Akt were from Cell Signaling Technologies Inc.

##### Directed Evolution

The DT40 chicken B cell line AID^R^CL4 (22) was grown in RPMI 1640 with 7% fetal bovine serum and 3% chicken serum at 37 °C and 5% CO_2_. Transfections were performed by electroporation in 0.4-cm cuvettes using a Gene Pulser (Bio-Rad) at 250 V and 950 microfarads and stable transfectants selected with puromycin. Transfected clones in which the Tie2 construct had integrated into the rearranged Ig locus were identified by PCR as described previously (22). Expression was confirmed by immunoblotting for the epitope tag, and Tie2 ectodomain and surface expression were confirmed by immunostaining of nonpermeabilized cells.

For ligand binding and fluorescence-activated cell sorting, between 50 and 100 million DT40 cells were washed at room temperature in phosphate-buffered saline containing 10% fetal bovine serum (PBS/FCS) and incubated with the appropriate ligands at 1 nm final concentration for 30 min at room temperature. Cells were recovered by centrifugation (250 × *g* for 3 min) at 4 °C, washed with ice-cold PBS/FCS, and then stained with anti-Ang1 in PBS/FCS at 4 °C for 20 min. Cells were then collected by centrifugation, washed in PBS/FCS, and stained with anti-FLAG-FITC, fluorescent anti-His_6_ or fluorescently labeled streptavidin (for biotinylated Ang2 detection), and fluorescently labeled secondary antibodies, as appropriate, in PBS/FCS at 4 °C for 20 min. After collecting by centrifugation and washing in ice-cold PBS/FCS, the stained cells were resuspended in PBS/FCS and kept on ice for FACS. The windows for selection were as indicated under “Results.” Sorted cells were recovered directly into culture medium at room temperature for further growth. Depending on the number of cells recovered, the times required between sorts to grow to 50–100 million cells were between 7 and 14 days. Cells were grown and sorted repeatedly as described under “Results.”

To sequence the Tie2 surface expression construct exogenously expressed in the DT40 cells, genomic DNA was prepared from DT40 cells using a Puregene DNA isolation kit (Qiagen). The Tie2 ectodomain insert was amplified by PCR, cloned into a bacterial sequencing plasmid using the TA cloning protocol (Invitrogen), and transformed into *Escherichia coli*. Colonies were picked at random, and plasmids were sequenced.

##### Expression of Soluble Ectodomains

For expression in Hek293 cells, cDNA encoding wild-type Tie2 ectodomain (1–442) was subcloned into pcDNA 3.1 upstream of a human Fc tag and C-terminal His_6_ sequence (kindly supplied by Dr Richard Kammerer). Site-directed mutagenesis was used to modify this wild-type sequence to correspond to the evolved mutants. Site-directed mutagenesis was performed essentially using the QuikChange protocol (Agilent Technologies) and confirmed by sequencing.

Soluble ectodomain-Fc fusion proteins were obtained by transfection of HEK293 cells in suspension using polyethylenimine (23), and cells were grown for 3–4 days to allow the fusion proteins to accumulate in the medium. Debris was removed from medium by centrifugation, and fusion protein was purified by nickel-nitrilotriacetic acid chromatography (Qiagen) followed by buffer exchange into Tris-buffered saline containing 10% glycerol. Protein concentrations were determined by Bradford assay. Proteins were stored at 4 °C.

##### Binding Assays

ELISA assays were performed in 96-well plates in which 5 μg/ml Ang1 or Ang2 was immobilized. Following blocking with TBS containing 1 mg/ml BSA and 0.1% Triton-X 100, different concentrations of fusion protein were allowed to bind for 1 h and, after washing, bound fusion proteins were detected with anti-Tie2 ectodomain antibodies followed by peroxidase-conjugated secondary antibody and colorimetric quantification. For quantitative comparison, data were fitted to saturation binding curves by nonlinear regression using the Prism 6 software (GraphPad Software Inc). Concentrations of ectodomains required for half-maximal binding were derived from the binding curves. In each ELISA experiment, wild-type binding was also performed, allowing derivation of maximum binding relative to that of wild-type ectodomain.

##### Cellular Assays

The endothelial cell line EA.hy926 was cultured in DMEM containing 10% fetal bovine serum at 37 °C and 5% CO_2_. Cells were quiesced by incubation in serum-free medium before activation with Ang1, in the absence or presence of Ang2, with or without 25 μg/ml wild-type or evolved ectodomain-Fc for 30 min. After washing, cells were lysed, and equal amounts of cellular proteins were resolved by SDS-PAGE before detection of phospho-Ser-473-Akt and total Akt by immunoblotting. Migration assays were performed in Transwell tissue culture wells containing 8-μm pore size inserts (BD Biosciences, Oxford, UK). Serum-free medium containing 250 μg/ml BSA together with Ang1 or Ang2, in the absence or presence of soluble ectodomain-Fc fusion protein, was placed in the lower chamber of the wells. 10^5^ endothelial cells, in serum-free medium containing 250 μg/ml BSA, were placed in the upper chambers, and cells were allowed to migrate for 4 h at 37 °C. Cells on the upper surface were gently removed with a cotton bud, and the membrane was fixed in 4% formaldehyde. Membranes were washed in PBS, and nuclei were stained with 4′,6-diamidino-2-phenylindole (0.1 μg/ml). Membranes were mounted in glycerol, and the numbers of cells migrating through the membrane were counted in five random fields on the underside of each insert membrane.

## RESULTS

**FIGURE 1.**
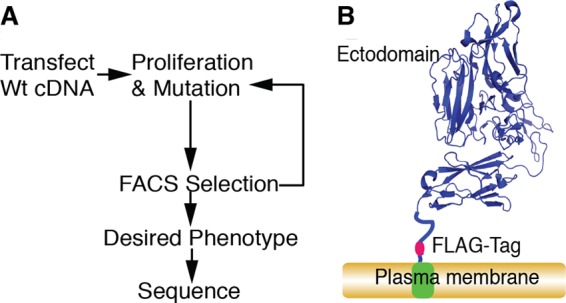
**Directed evolution of a ligand-specific Tie2 receptor ectodomain.** *A*, strategy for directed evolution in hypermutating B cells. *B*, schematic representation of the surface expression construct incorporating residues 1–442 of Tie2 (modeled on Protein Data Bank (PDB) accession number 2GY7 (26)) and used for directed evolution.

**FIGURE 2.**
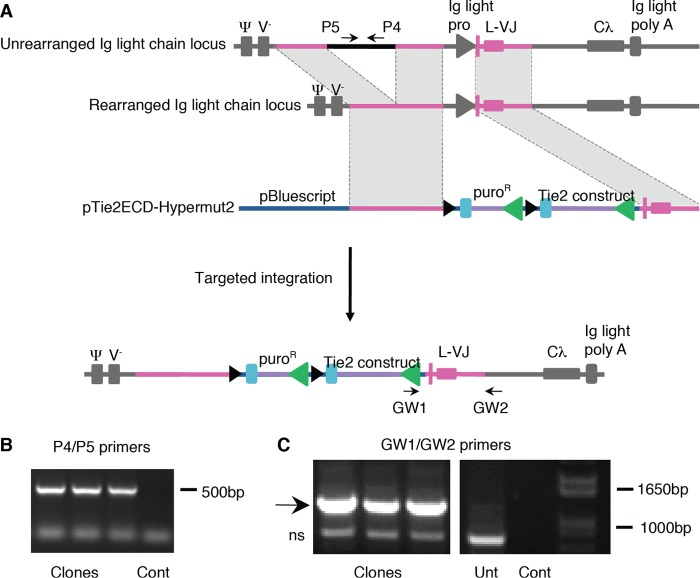
**Targeted integration of Tie2-Hypermut2 into DT40 Ig locus.** *A*, schematic representation of unrearranged and rearranged Ig locus and Tie2-Hypermut2 with regions of homology (*pink*) and integrated construct. The positions of primers P4 and P5 are indicated. PCR of DT40 genomic DNA from transfectants with P4/P5 amplify a 493-bp segment, as seen in *panel B* for three representative clones, confirming that integration has not occurred in the unrearranged locus. PCR amplification of genomic DNA from transfected DT40 with primers GW1/GW2 amplify a 1189-bp segment when Tie2-Hypermut2 integrates into rearranged locus, as is shown for three clones in *panel C*. Control amplifications (*Cont*) without DNA and amplifications from untransfected DT40 genomic DNA (*Unt*) are also shown; *ns* indicates a nonspecific amplification product.

**FIGURE 3.**
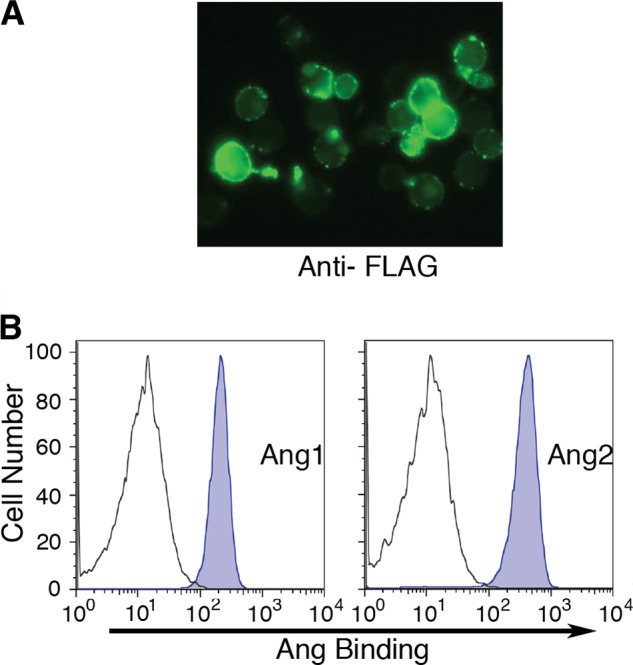
**Expression and binding of ectodomain on DT40 cell surface.** *A*, anti-FLAG immunofluorescent staining of DT40 cells transfected with surface expression construct. *B*, flow cytometry of DT40 cells expressing Tie2 ectodomain. Untransfected (*gray open plot*) and transfected (*blue-shaded plot*) cells were allowed to bind His_6_-tagged 1 nm Ang1 or Ang2 for 30 min before staining with anti-His and fluorescent secondary antibody.

Combining cell surface display with the ability of certain B cell lines to diversify genes targeted to immunoglobulin loci could provide a powerful strategy for directed evolution of protein binding and other functions (Fig. 1*A*). Therefore we used this approach to seek to evolve a Tie2 ectodomain that preferentially binds Ang2 over the protective ligand Ang1, with which it shares more than 70% amino acid sequence identity in its receptor-binding domain. To do this, a cDNA sequence encoding residues 1–442 of the Tie2 ectodomain together with a linker sequence, epitope tag, and PDGF receptor transmembrane domain was constructed for surface expression of the ectodomain in B-cells (Fig. 1*B*). Previous work has shown that angiopoietin binding only requires residues 23–210 of Tie2 (24). However, it is known that in other proteins, mutations at sites remote from the interaction domain can often affect binding ability (25) and, for this reason, we included additional portions of Tie2 ectodomain beyond residue 210 in our directed evolution strategy. The cDNA construct was cloned into a vector, pHypermut2, we previously developed (22) for targeted integration into the Ig locus of the chicken cell line DT40. Chicken B cells normally diversify their Ig loci by a combination of gene conversion, using an array of upstream IgV pseudogene segments, and by untemplated somatic hypermutation. We used a variant of DT40 in which the IgV pseudogene donors have been deleted and which diversifies its rearranged Ig light chain by hypermutation (22). Insertion of Tie2 transgene in the Ig locus of this cell line in the absence of nearby Tie2 homologous sequences was expected to lead to hypermutation of the Tie2 transgene. Stably transfected clones were selected for expression of the construct from the rearranged IgV locus by PCR (Fig. 2). Surface expression was verified by anti-FLAG immunofluorescence (Fig. 3*A*). We also confirmed that the ectodomain was competent to bind Ang1 and Ang2 by flow cytometry (Fig. 3*B*).

**FIGURE 4.**
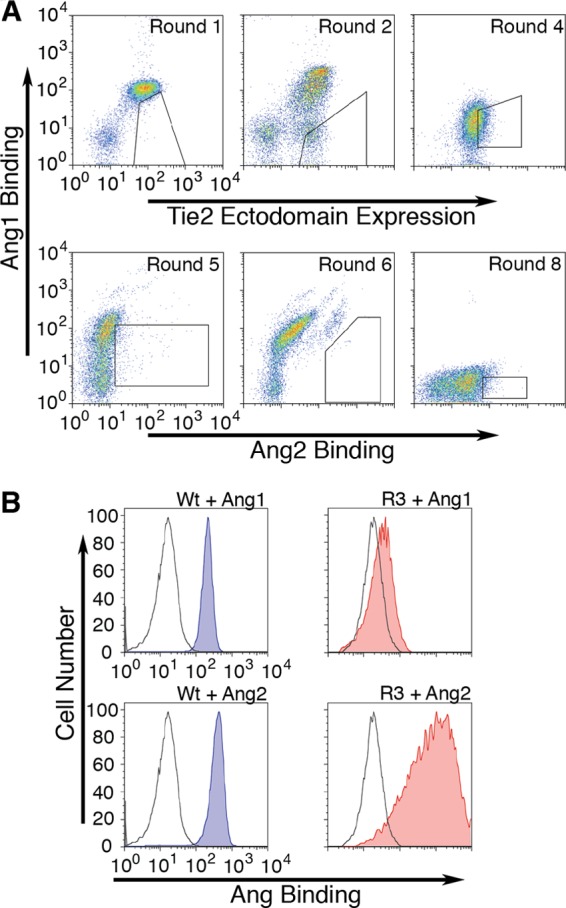
**Evolution of ligand-specific Tie2 ectodomain.** *A*, *upper panels*, FACS plots of DT40 cells following incubation with 1 nm Ang1 and staining with anti-Ang1 and fluorescent secondary antibody together with fluorescent anti-FLAG (*Tie2 Ectodomain Expression*). *Polygons* indicate the gates used to select the cells at each round. Cells from sort round 4 were then incubated with 1 nm Ang1 and 1 nm biotinylated Ang2, and binding was detected with anti-Ang1/fluorescent secondary antibody and fluorescently labeled streptavidin (*lower panels*). Cells were selected for highest Ang2 binding. After a total of 8 rounds of sorting (*lower panel*, *right*), cells were selected for sequencing as indicated by the *polygon. B*, comparison of DT40 cells expressing wild-type receptor (*blue-shaded plots*) with the evolved R3 (*red-shaded plots*) population of cells for binding of 1 nm Ang1 and Ang2. *Gray open plots* show fluorescence following staining in the absence of ligand for each population of cells.

To evolve Tie2 to preferentially bind Ang2, we used a two-stage strategy, first aiming to decrease the ability of the ectodomain to bind Ang1 and then to test, and if necessary, increase Ang2 binding while maintaining low Ang1 binding. For the first stage, cells were incubated with 1 nm Ang1, and bound ligand was detected by anti-Ang1 and phycoerythrin/Cy5-conjugated secondary antibody as detailed under “Experimental Procedures.” Expression of Tie2 ectodomain construct was monitored simultaneously by staining with FITC-conjugated anti-FLAG (Fig. 4*A*). Cells positive for expression of the Tie2 ectodomain and with the lowest Ang1 binding were selected by FACS and expanded (Fig. 4*A*). The sort windows used for each selection are shown in Fig. 4*A*. Typically between 50 and 100 million cells were screened at each sorting. Following FACS, recovered cells were re-expanded to allow for another round of sorting, typically for 7–14 days, depending on the number of cells obtained at FACS. After four iterations of selection and expansion, a population of cells with decreased Ang1 binding as compared with parental cells was obtained. We then changed the selection strategy to ensure robust Ang2 binding. We incubated the round 4 cells with 1 nm Ang1 and 1 nm biotinylated Ang2 and monitored binding of the two ligands with anti-Ang1 and fluorescent secondary antibody and fluorescently labeled streptavidin (Fig. 4*A*, *lower plots*). Cells with highest Ang2 binding that retained low Ang1 binding were selected by FACS using the sort windows indicated in Fig. 4*A*. After four rounds of this selection and expansion regime, a population of cells with apparent preferential binding to Ang2 was evolved, which we designated R3.

Direct comparison of parental and R3 cells for their ability to bind Ang1 and Ang2 was performed for each of the ligands (Fig. 4*B*). Cells in the R3 population appeared only able to bind Ang2 and had negligible Ang1 binding, whereas parental cells expressing wild-type Tie2 were able to bind both ligands.

**FIGURE 5.**
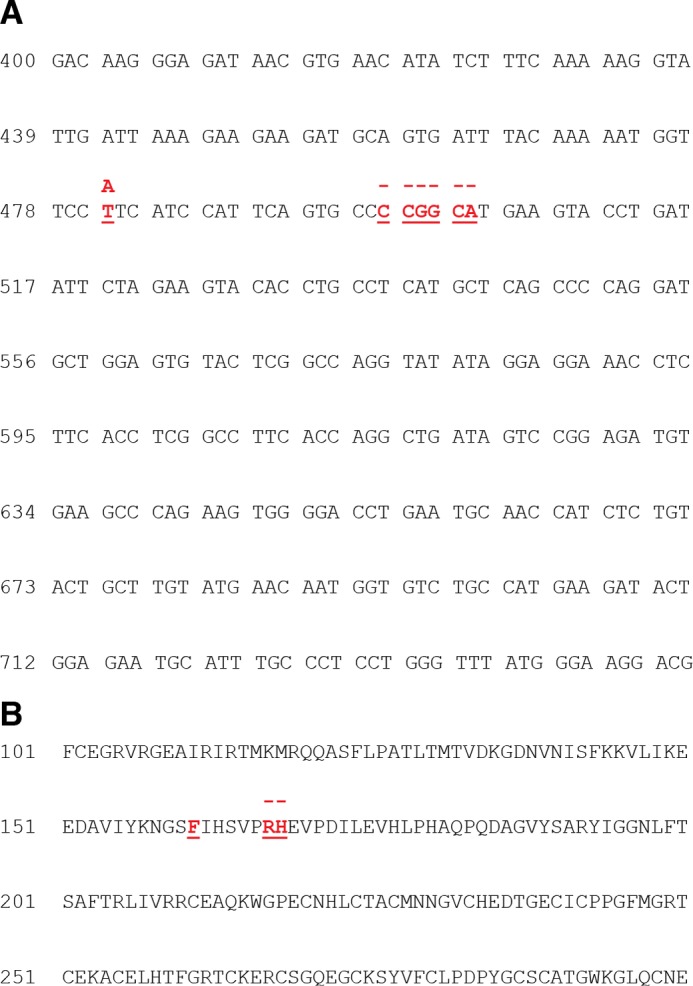
**Three amino acid changes switch binding specificity of Tie2.** Genomic DNA was prepared from the R3 population and used for amplification of DNA encoding Tie2 ectodomain. 10 randomly selected colonies were sequenced following transformation into *E. coli. A*, the nucleotide changes common to the R3 sequences are colored *red*, and in the substituted nucleotide shown above, a *dash* indicates a deletion. *B*, amino acid changes resulting from mutations are shown in *red*.

**FIGURE 6.**
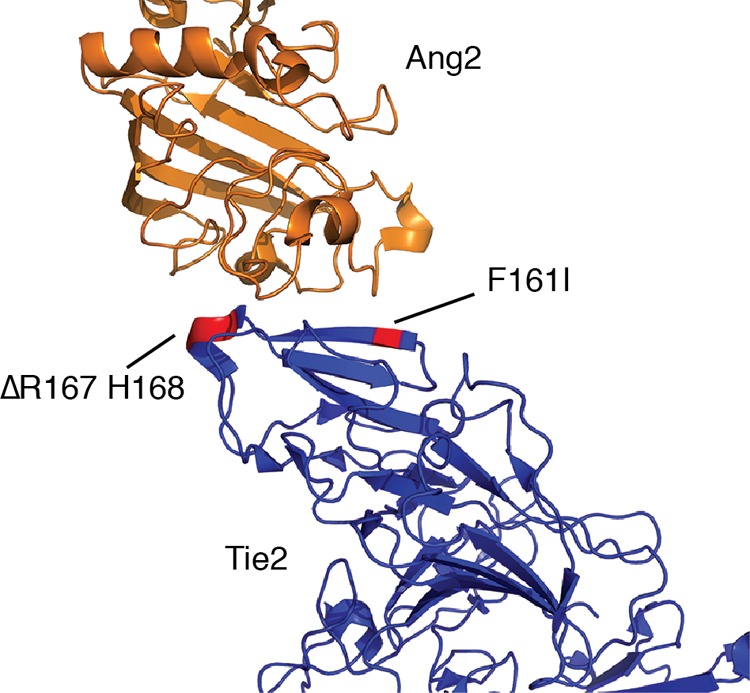
**Amino acid changes occur at the ligand-binding interface.** The positions of the amino acid changes in the evolved ectodomain are indicated on the structure of Tie2 in complex with Ang2 (PDB accession number 2GY7 (26)). The F161I substitution is positioned on a β sheet, and the Arg-167/His-168 deletion occurs on a turn at the receptor-ligand interface.

We next obtained sequences encoding the ectodomain that was expressed on the cells with preferential Ang2 binding (Fig. 5*A*). 10 sequences were determined, and all had a common set of changes; specifically, Phe-161 was replaced by Ile, and there was a tandem deletion of Arg-167 and His-168 (Fig. 5*B*). The F161I substitution was the result of a single nucleotide change in the Phe-161 codon from TTC to ATC. The Arg-167/His-168 double deletion resulted from loss of the final Cys of codon Pro-166 together with the CGG encoding Arg-167 and the first two nucleotides, CA, of codon His-168. This created a new codon for Pro-166, CCT, and removal of Arg-167/His-168 (Fig. 5). In addition to these changes, a number of other mutations were found in the R3 population; however, none of these were present in all sequences. Interestingly, examination of the published structure of Tie2 ectodomain revealed that both the F161I substitution and the double Arg-167/His-168 deletion occur at the binding interface of the receptor, as illustrated in Fig. 6 for the Tie2-Ang2 complex (26).

**FIGURE 7.**
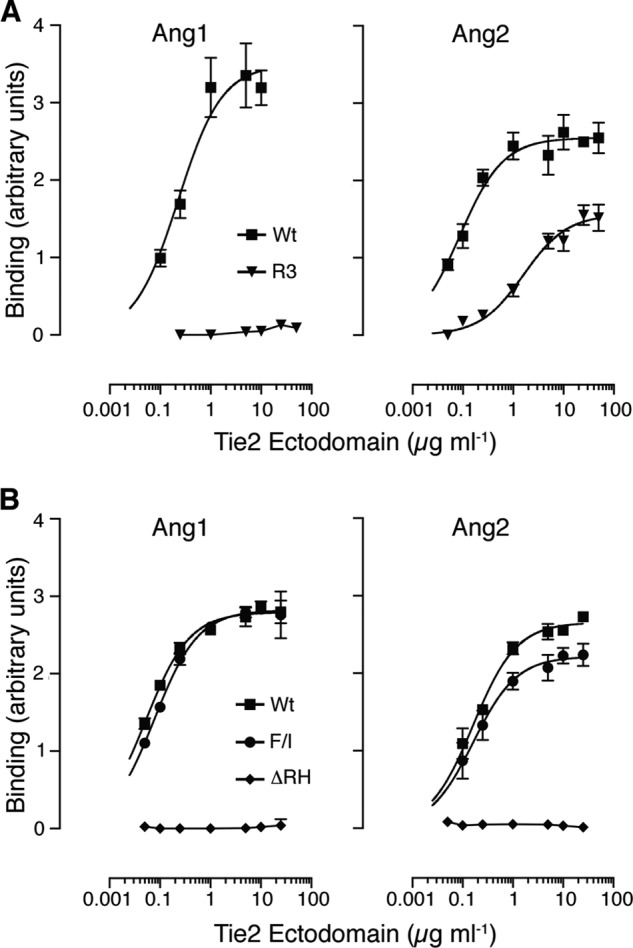
**Evolved ectodomain binds specifically to Ang2.** *A*, secreted wild-type (*Wt*; *squares*) and evolved (*R3*; *inverted triangle*) ectodomains were purified following expression in HEK293 cells and analyzed for binding to immobilized Ang1 or Ang2 by ELISA, as indicated. *B*, secreted wild-type ectodomain (*Wt*) and ectodomains with either F161I substitution (*F/I*; *circles*) or the double Arg-167/His-168 deletion (Δ*RH*; *diamonds*) were expressed, purified, and analyzed for binding to immobilized Ang1 or Ang2 by ELISA, as indicated. Data are shown as means and standard deviations from a single experiment with triplicate determinations. All binding assays were repeated at least three times, a representative experiment is shown in each panel, and the data are summarized in Table 1.

To analyze the binding characteristics of the evolved ectodomain in more detail, we constructed the wild-type ectodomain (residues 1–442) with a carboxyl-terminal Fc tag and introduced the F161I and ΔArg-167/His-168 into this sequence by site-directed mutagenesis. Wild-type and R3 ectodomains were expressed in HEK293 cells as secreted soluble proteins of ∼80 kDa and purified (data not shown). Binding of the wild-type ectodomain and the evolved ectodomain to Ang1 and Ang2 was examined using ELISA assays (Fig. 7*A*). As expected, the wild-type ectodomain bound both ligands (Fig. 7*A,* Table 1). In contrast, the evolved ectodomain was able to bind Ang2 but showed negligible Ang1 binding (Fig. 7*A*, Table 1), consistent with our observation on this variant ectodomain when expressed on the cell surface (Fig. 4*B*). The Ang2 binding ability of the evolved ectodomain was not as high as that of wild-type ectodomain, with lower maximal binding and a higher concentration required for half-maximal binding (Table 1). Nevertheless, the evolved protein clearly demonstrates preferential Ang2 binding. It was surprising to us that changes at only three residues caused such a dramatic switch in the binding specificity of the receptor ectodomain.

**TABLE 1:** **Relative binding of ectodomain mutants** Wild-type (WT) and evolved (R3) ectodomains, as well as ectodomains with F161I (F/I) substitution or Arg-167 and His-168 (ΔRH) deletion were expressed, purified, and analyzed for binding to immobilized Ang1 or Ang2 by ELISA (Fig. 7). Maximum binding relative to WT ectodomain and concentrations (conc.) of ectodomains required for half-maximal binding were derived from saturation binding curves fitted by nonlinear regression (— indicates that lack of measurable binding precludes determination of half maximal binding concentration). Data are means and S.E. for at least three independent experiments (* indicates *p* < 0.01 compared with wild-type, Student's *t* test).

	Ang1		Ang2	
	Max binding (% of WT)	Half max binding conc.	Max binding (% of WT)	Half max binding conc.
		μ*g/ml*		μ*g/ml*
WT	100.00	0.04 ± 0.01	100.00	0.25 ± 0.09
R3	3.00 ± 3.29*	—	59.40 ± 5.28*	6.98 ± 2.12*
F/I	97.84 ± 2.63	0.06 ± 0.02	90.48 ± 4.62	0.29 ± 0.17
ΔRH	0.29 ± 0.16*	—	0.58 ± 0.58*	—

We were interested in examining the individual effects of the F161I substitution and double ΔArg-167/His-168 deletion on binding. We therefore constructed wild-type Fc soluble ectodomain with F161I substitution or ΔArg-167/His-168 changes and tested Ang1 and Ang2 binding in ELISA assays. There was no significant difference between wild-type and F161I ectodomains in binding to Ang1 (Fig. 7*B*, Table 1). Similarly, wild-type and F161I ectodomains both bound equally well to Ang2 (Fig. 7*B*, Table 1). In contrast, deletion of Arg-167/His-168 completely abolished ectodomain binding to Ang1 and Ang2 (Fig. 7*B,* Table 1). This was an unexpected result and showed that the changes introduced by the directed evolution act in a uniquely combinatorial rather than an additive way to switch the binding specificity of the ectodomain.

**FIGURE 8.**
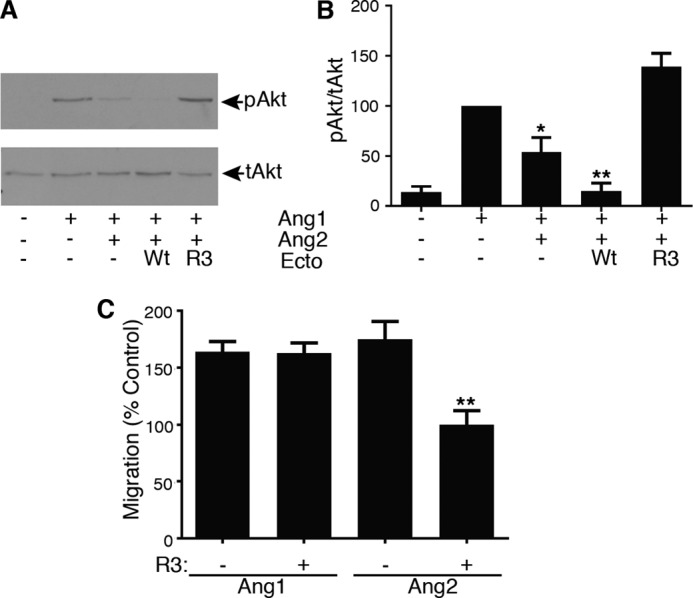
**Evolved ectodomain blocks the effects of Ang2 on endothelial cells.** *A*, the antagonistic effects of Ang2 on Ang1 activation of Akt phosphorylation were tested in the endothelial cell line EA.hy926. Cells were activated with 0.7 nm Ang1 in the absence and presence of 3 nm Ang2 and 25 μg/ml wild-type (*Wt*) or R3 ectodomain (*Ecto*) for 30 min before cell lysis, gel electrophoresis, and immunoblotting with antibodies recognizing Akt phosphorylated on Ser-473 (*pAkt*) or total Akt (*tAkt*) as indicated. *B*, Akt phosphorylation was quantified by densitometric scanning of blots from four independent experiments, normalized to total Akt, and expressed as the percentage of Ang1 effect. Data are means and S.E. (*, *p* < 0.05, **, *p* < 0.01 *versus* Ang1, one-way analysis of variance followed by Tukey's analysis). *C*, effects of evolved ectodomain were tested on migration induced by angiopoietins. Migration of endothelial cells in response to high concentrations of Ang2 (15 nm) was inhibited by R3 ectodomain, whereas this mutant ectodomain did not affect migration in response to Ang1 (0.7 nm). Data are shown as the percentage of that in the absence of angiopoietin and presented as means and S.E. for four independent experiments (**, *p* < 0.01 *versus* angiopoietin, one way analysis of variance followed by Tukey's analysis).

To test the effects of R3 ectodomain on the cellular actions of Ang2, we performed a signaling assay to examine its ability to interfere with Ang2 antagonism of Ang1 in the endothelial cell line EA.hy926. Endothelial cells challenged with Ang1 showed an activation of the signaling intermediate Akt and, consistent with the reported antagonist effects of Ang2, this was suppressed by Ang2 (Fig. 8, *A* and *B*). Inclusion of wild-type ectodomain with Ang1 plus Ang2 further suppressed Akt activation, as expected from the ability of this fusion protein to bind Ang1 as well as Ang2. However, when the evolved ectodomain was added with Ang1 and Ang2, the inhibitory effect of Ang2 was reversed and Akt activation restored (Fig. 8, *A* and *B*). To further examine the action of evolved ectodomain in a cellular context, we tested its effects on a functional activity in endothelial cells, specifically, endothelial migration in response to Ang1 and high concentrations of Ang2. Previously, high concentrations of Ang2 have been reported to have agonist activity in endothelial cells (1, 2). As shown in Fig. 8*C*, the addition of the evolved ectodomain did not affect Ang1-induced migration but blocked migration in response to Ang2. These data demonstrate that the evolved ectodomain can block the effects of Ang2 at both signaling and functional levels in endothelial cells without interfering with Ang1, as would be expected by its preference for binding Ang2 over Ang1.

## DISCUSSION

In this study, we have evolved a new form of Tie2 ectodomain with dramatically shifted binding specificity. This change in binding specificity results from a combination of just three amino acid changes at the binding interface with angiopoietins that would be unlikely to be identified by rational design. Soluble forms of this ectodomain bind Ang2 but not Ang1. Furthermore, the evolved ectodomain acts as a ligand trap in cellular assays, blocking Ang2 effects on endothelial cell signaling, and function without interfering with Ang1. In contrast to Ang2, Ang1 has important roles in vascular protection, so a molecule that can block Ang2 without interfering with Ang1 would have significant benefits for therapeutic use, particularly in conditions associated with inflammation. Our evolved ectodomain therefore is an attractive molecule for further development as a potential therapeutic.

The receptor-binding interfaces of Ang1 and Ang2 are very similar, and it was unexpected that only three amino acid changes in the Tie2 ectodomain are sufficient to change binding specificity. Crystal structures of Ang2 in complex with Tie2 (PDB 2GY5), and Ang1 in complex with Tie2 (PDB 4K0V), have been reported, and the structures of both ligand-Tie2 complexes are very alike (26, 27). Examination of the crystal structure of Tie2 ectodomain bound to Ang2 shows that Phe-161 of Tie2 stacks with Phe-469 in Ang2 (26). The equivalent position in Ang1 is occupied by Leu-471. Substitution of Ile for Phe-161 in Tie2 therefore retains the hydrophobic character of this position but would negatively affect aromatic stacking between Phe-161 in Tie2 and Phe-469 in Ang2. Our finding that the F161I substitution did not affect relative binding to Ang1 and Ang2 suggests that aromatic stacking at this position does not make a significant contribution to differential binding.

Arg-167 in Tie2 forms a salt bridge with Asp-448 in Ang2 (26) and the corresponding Asp-450 in Ang1 (27). In the ectodomain, His-168 forms a hydrogen bond with Tyr-476 in Ang2 and also interacts with Pro-452, and both of these positions are conserved (Tyr-478 and Pro-454) in Ang1 (26). Thus it would be anticipated that loss of Arg-167/His-168 would affect both Ang1 and Ang2 binding in a similar manner, and indeed this double deletion did lead to loss of binding of both ligands (Fig. 7*A*). The lack of binding of Arg-167/His-168 Tie2 to Ang1 and Ang2 suggests that the hydrogen bonds and salt bridges contributed by these residues are important for stabilizing the ligand-receptor complexes. It is intriguing that the F161I substitution is able to rescue the Arg-167/His-168 deletion effect on Ang2 binding. This suggests that in the absence of Arg-167/His-168, the Ile at 161 can form favorable interactions with one or more residues in Ang2 that are not present, or prevented from interacting, in Ang1. This may occur if loss of Arg-167/His-168 and associated noncovalent bonds allows repositioning of ligands at the ectodomain interface, thereby permitting Ile-161 to make such an interaction with residue(s) in Ang2 and/or preventing such an interaction in Ang1. However, such a mechanism is speculative, and in future work, it will be important to determine the structure of the evolved ectodomain bound to Ang2 to define the basis for the ligand specificity of this ligand trap.

Our evolution of Tie2 ectodomain to discriminate between Ang1 and Ang2 has created an Ang2 ligand trap with potential for blocking the deleterious effects of the elevated Ang2 in conditions such as vascular inflammation, sepsis, and cancer. In addition, our study provides proof of principle for the use of cell surface display in combination with somatic hypermutation-driven gene diversification as a facile method for directed evolution of binding proteins. Because this method uses vertebrate B-cells as the expression system, it is readily applicable to proteins that may require the processing and post-translational modifications found in higher eukaryotes. This approach therefore may find general use for evolving receptor or ligand fragments with specific binding characteristics useful for therapeutic and other applications.

## Footnotes

2 The abbreviation used is:

Ang: angiopoietin.
